# Construction and validation of indicators and respective definitions for the nursing outcome Swallowing Status^1^

**DOI:** 10.1590/0104-1169.0377.2575

**Published:** 2015-07-03

**Authors:** Ana Railka de Souza Oliveira, Thelma Leite de Araujo, Emilia Campos de Carvalho, Alice Gabrielle de Sousa Costa, Tahissa Frota Cavalcante, Marcos Venícios de Oliveira Lopes

**Affiliations:** 2 Post-doctoral fellow, Escola de Enfermagem de Ribeirão Preto, Universidade de São Paulo, PAHO/WHO Collaborating Centre for Nursing Research Development, Ribeirão Preto, SP, Brazil. Scholarship holder from Conselho Nacional de Desenvolvimento Científico e Tecnológico (CNPq), Brazil; 3 PhD, Full Professor, Departamento de Enfermagem, Universidade Federal do Ceará, Fortaleza, CE, Brazil; 4 PhD, Full Professor, Escola de Enfermagem de Ribeirão Preto, Universidade de São Paulo, PAHO/WHO Collaborating Centre for Nursing Research Development, Ribeirão Preto, SP, Brazil; 5 PhD, Professor, Faculdade Grande Fortaleza, Fortaleza, CE, Brazil; 6 PhD, Professor, Departamento de Enfermagem, Universidade da Integração Internacional da Lusofonia Afro-Brasileira, Redenção, CE, Brazil; 7 PhD, Associate Professor, Departamento de Enfermagem, Universidade Federal do Ceará, Fortaleza, CE, Brasil

**Keywords:** Aerophagy, Stroke, Nursing Assessment, Validation Studies

## Abstract

**OBJECTIVE:**

to develop indicators for the nursing outcome Swallowing Status and the respective conceptual and operational definitions validated by experts and in a clinical setting among patients after having experienced a stroke.

**METHOD:**

methodological study with concept analysis and content and clinical validations. The Content Validation Index was verified for the scores assigned by 11 experts to indicators. Two pairs of nurses assessed 81 patients during the clinical validation: one pair used an instrument with definitions and the other used an instrument without definitions. The resulting assessments were compared using Intraclass Correlation Coefficient, Friedman's test, and Minimal Important Difference calculation.

**RESULTS:**

All the indicators, with the exception of the indicator Ability to bring food to mouth, presented Content Validation Index above 0.80. The pair using the instrument with definitions presented an Intraclass Correlation Coefficient above 0.80 for all the indicators and similarity was found in all the assessments, according to the Minimal Important Difference calculation. The pair using the instrument without definitions presented a low coefficient (ρ<0.75) for all the indicators.

**CONCLUSION:**

the results showed that greater uniformity and accuracy was achieved by the pair of nurses using the conceptual and operational definitions for the indicators of the nursing outcome Swallowing Status.

## Introduction

Patients commonly experience changes in swallowing after a stroke. There are records^1^ that in acute stages of the disease, this condition affects more than 50% of the patients, reducing to about 44% in the rehabilitation phase. This condition is associated with increased mortality and dependence (institutionalization). 

Therefore, dysphagia appears as an inability that contributes to loss of functionality and independence in eating, imposing the risk of malnutrition and aspiration pneumonia. Hence, it directly or indirectly affects life in many ways, leading to implications that include not only problems of a biological nature, but also psychological and social problems^2^. In this context, it is essential that nurses be able to assess patients affected by a stroke who experience changes in swallowing, to prevent complications, monitor clinical indicators and to assess the efficacy of nursing interventions.

Various specific nursing classification systems can be used to assess clinical indicators, such as the Nursing Care Report Card for Acute care, the Quality Health Outcomes Model, the OMAHA System, Home Health Care Classification (HHCC), The Patient Care Data Set, The Outcome Assessment Information Set (OASIS), the International Classification for Nursing Practice (ICNP(r)) and the Nursing Outcome Classification (NOC)^3^, which was adopted in this study.

The NOC presents the following nursing outcomes to assess swallowing functions: Swallowing Status; Swallowing Status: oral phase; Swallowing Status: pharyngeal phase; Swallowing Status: esophageal phase; and Aspiration prevention. Note that only the nursing outcome Swallowing Status contains essential indicators to assess the entire swallowing process.

According to the NOC, version 2010^4^ adopted for this study, Swallowing Status refers to the "safe passage of fluids and/or solids from the mouth to the stomach." It includes the following indicators: Maintains food in mouth, Handles oral secretions, Saliva production, Chewing ability, Delivery of bolus to hypopharynx timed with swallow reflex; Ability to clear oral cavity; Timely bolus formation; Number of swallows appropriate for bolus size/texture; Meal duration with respect to amount consumed; Timely swallow reflex; Maintains neutral head and trunk position; Food acceptance; Swallow study findings; Changes in voice quality; Choking; Coughing; Gagging; Increased swallow effort; Gastric reflux; and Discomfort with swallowing.

The indicators presented by the NOC taxonomy for each outcome are intended to help nurses define the health condition of patients; however, they are not sufficient to reliably estimate one's real health condition, as each individual examining or observing a patient assigns scores according to his/her perception^5^.

Hence, the development of conceptual and operational definitions is recommended for each of the indicators. It is believed that by using scales with definitions that enable continuous monitoring of patients for a given period of time, health professionals are permitted rapidly and reliably to identify changes in a patient's condition, thus ensuring greater effectiveness in the implementation of early interventions, in addition to ensuring more accurate reassessments.

This study's objectives included developing and performing expert and clinical validation of indicators for the nursing outcome Swallowing Status and the respective conceptual and operational definitions among patients affected by a stroke. 

## Method

This methodological study's aim was to develop, validate and assess instruments to improve reliability and validity^6^. The selected nursing outcome was submitted to Content Analysis, Content Validation and Clinical Validation, which contributed to its improvement.

An integrative review was performed for the Conceptual Analysis, which enabled finding papers addressing this topic, in addition to dissertations, theses and books. The NOC indicators were revised and conceptual and operational definitions were developed for each indicator. Additionally, for each magnitude, that is, for each of the five points on the Likert scale, an operational definition was established to help nurses during assessments.

During the Content Validation stage, the nursing outcome with its indicators and respective conceptual and operational definitions was submitted to 11 judges. This number of judges was established according to psychometric recommendations of a minimum of six judges^7^.

The judges examined the relevance and clarity of each indicator and respective definition according to the following: -1 (inappropriate definition/indicator), 0 (somewhat appropriate definition/indicator), and +1 (appropriate definition/indicator). Based on the scores assigned by the judges, the Content Validation Index (CVI) was computed with a cut-off point of 0.80^8^. There was one indicator that did not reach this cut-off point, but was added nevertheless because a theoretical review showed its importance for clinical practice and also due to a lack of theory-based grounds for its exclusion.

Note that, at this point, the judges had the liberty to suggest changes concerning the names of the indicators, on their grouping or exclusion. The researchers, in turn, examined each suggestion and justification and determined whether they were pertinent or not, based on personal knowledge and according to what is recommended in the literature.

The population addressed in the Clinical Validation was composed of inpatients with a diagnosis of a stroke. Inclusion criteria were: being at least 18 years old; being conscious and able to provide information, or otherwise being accompanied by a caregiver able to provide information, concerning the patient's health condition. Exclusion criteria were adopted according to recommendations found in the literature^9^: presenting, at the time of data collection, hemodynamic instability with a risk of death or using invasive mechanical ventilation or enteral tubes, because these raise the risk of respiratory aspiration.

Data were collected between January and July 2013 in a hospital ward where care was provided exclusively to patients in the acute and sub-acute phase of stroke. Given a lack of uniformity in the definition of sample sizes provided in validation studies, which range from 5 to 20 participants for each of the items on a scale, we opted to work with at least eight patients with stroke for each of the indicators^10^. Note that only the nine indicators validated by the experts, and the one considered to be pertinent by the researchers, which totaled ten indicators, were clinically validated.

After the initial assessment of patients, two pairs of nurses clinically assessed the indicators of the nursing outcome Swallowing Status. The first pair used the instrument previously developed with definitions. The second pair applied the same instrument with the revised indicators, but without the definitions according to the model presented by the NOC.

The following criteria were used to select the nurses: professional experience of at least one year in providing care to patients who suffered a stroke or with dysphagia or in critical care units or being a member of research groups studying diagnoses, interventions and nursing outcomes.

Additionally, all the nurses received 20 hours of training in which the NOC nursing outcomes were discussed together with the indicators of the outcome Swallowing Status and their respective definitions. Note that the pairs equally participated in the training, but that the pair receiving the instrument without definitions were not provided the definitions developed for Swallowing Status in their training.

Before initiating the clinical validation, a pre-test was performed with four patients affected by a stroke, who were not included in the study's final sample. This pre-test enabled the researchers to verify the time necessary for collecting data and to implement necessary adjustments.

After the pre-test and training program, amendments were made to the instrument, such as replacing the names of the indicators: from "Assessment of mastication structures" to "Integrity of structures involved in mastication" and from "Oral cavity cleaning" to "Oral cavity emptying". The researchers made these changes to ease the understanding of evaluators and because these changes were restricted to the names of the indicators.

The pair of nurses using the instrument with definitions simultaneously assessed the patients during the assessment of the nursing outcomes. The assessment of items that involved the handling of some oral and/or neck structures, however, was individually performed. The pair of nurses using the instrument without definitions addressed the patient separately to avoid influencing how each professional would assess the indicators^5^.

Note that the pairs addressed the patient separately and did not talk to each other, so as to ensure that the assessments were independently performed. The researchers supervised all the assessments and accompanied data collection to ensure methodological rigor was maintained.

Data were compiled in Excel 8.0, processed and analyzed using SPSS version 20.0, and R version 2.10. To verify the reproducibility of assessments performed by the pairs of nurses, we estimated the intraclass correlation coefficient (ICC). In this case, assessment was performed intra-group; that is, correlation was compared between the nurses who used the same instrument. The idea was to assess the degree of relationship between the nurses using the same instrument, both between those using the conceptual and operational definitions and between those not using the definitions.

ICC verified similarity between assessments and whether the measurements increased or decreased together. ICC values close to 1 indicated greater agreement between assessments. 

We next used the Friedman test to verify the difference of medians among the four nurses. In the case of statistically significant differences, we proceeded to *post hoc* analysis using the method of minimal important difference (MID). This calculation estimates a minimum value between the differences of ranks and then enables a pairwise comparison between the scores of each result among the nurses; i.e., after computing the general MID, the values between the differences of average ranks for the score assigned by each nurse in both the pair using definitions and the pair not using definitions were computed. 

When the value between the differences of average ranks of two evaluators was greater than the general MID (calculated), we consider there to be a significant statistical difference between the assessments of those two evaluators. If the difference between the average ranks was below the calculated MID, we considered there to be no statistical difference between the assessments. This value was compared intra and intergroup. Note that, for all the tests, the level of significance adopted was 5% (p≤0.05).

All ethical recommendations concerning research involving human subjects were complied with. Data collection was initiated only after approval was obtained from the Institutional Review Board at the Federal University of Ceará (Protocol No. 215.770).

## Results

**Figure 1.**
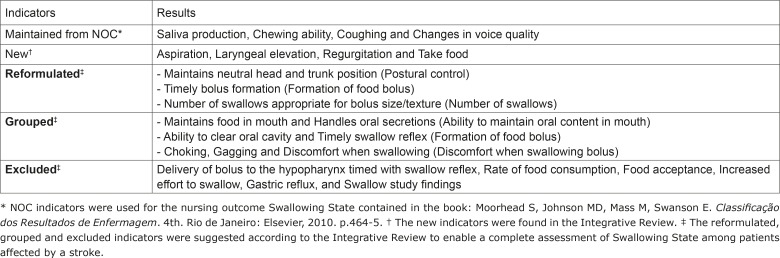
Summary of results of Concept Analysis for the nursing outcome Swallowing Status for patients after stroke.

The Integrative Review and Conceptual Analysis enabled refining the nursing outcome, which began by presenting 14 possible indicators to be assessed at bedside and the development of their respective conceptual and operational definitions (Figure 1).

The 14 indicators resulting from the Content Analysis were submitted to Content Validation, performed by ten nurses and one speech therapist. These professionals mastered theory and practice concerning the subject of swallowing and in providing care to patients affected by a stroke. Two of these doctorates and nine had Master's degrees. The healthcare workers who were nurses also had knowledge of nursing taxonomies. 

Content validation resulted in the maintenance of three indicators (Coughing, Laryngeal elevation and Regurgitation); three groupings (Maintains oral content in mouth and saliva secretion, which became Ability to maintain oral content in mouth; Formation of food bolus, Number of swallows, and Ability to clean oral cavity, which became Cleaning of oral cavity; and Aspiration and Change of voice quality, which was renamed Respiratory Aspiration); adaptation of the title of four indicators: Take food (Ability to bring food to mouth), Ability to chew (Structural assessment of mastication), Postural control (Postural control of head and neck in relation to body) and Discomfort when swallowing bolus, according to the results presented in Table 1.

**Table 1.** Content validity index of names of indicators, conceptual and operational definitions in relation to clarity and relevance criteria. Fortaleza, CE, Brazil, 2013

Indicators*	Name		Conceptual definition		Operational definition	
	Clarity	Relevance	Clarity	Relevance	Clarity	Relevance
1	0.45	0.63	0.45	0.64	0.45	0.64
2	0.91	0.82	0.91	0.91	0.91	0.91
3	0.82	0.64	0.73	0.64	0.73	0.64
4	0.82	0.91	0.91	0.91	0.91	0.91
5	0.91	0.82	0.73	0.73	0.73	0.73
6	0.73	0.73	0.45	0.45	0.45	0.45
7	0.36	0.36	0.54	0.64	0.54	0.64
8	0.73	0.91	0.91	0.91	0.91	0.91
9	0.45	0.36	0.36	0.09	0.36	0.09
10	1.00	1.00	0.82	1.00	0.82	1.00
11	0.82	0.72	0.82	0.73	0.82	0.73
12	0.91	1.00	0.82	0.91	0.82	0.91
13	0.72	0.82	0.82	0.82	0.82	0.82
14	1.00	0.91	0.91	0.91	0.91	0.91

*Indicators: 1. Take food; 2. Ability to chew; 3. Saliva secretion; 4. Ability to maintain oral content in mouth; 5. Formation of food bolus; 6. Number of swallows; 7. Ability to clean oral cavity; 8. Postural control; 9. Change of voice; 10. Cough; 11. Regurgitation; 12. Discomfort when swallowing bolus; 13. Laryngeal elevation; 14. Aspiration.

Note that even though the indicator Ability to bring food to mouth presented low CVI values (0.54), it was not excluded due to its consistency with the literature. For this reason, we decided to verify its clinical validation. The remaining indicators presented CVI values above 0.80 and their groupings were based on the literature and accepted by the researchers (Table 1). The new instrument with 10 indicators and their respective definitions was submitted to clinical validation

A total of 81 patients who suffered a stroke participated in the Clinical Validation, most of whom were male (58.0%), had a partner (65.4%); they were aged 56.3 years old on average (SD=14.6), ranging from 24 to 90 years old. According to the results, half of the sample was younger than 59 years old and attended school for up to five years. 87.7% had suffered an ischemic stroke; 27.1% had the right hemisphere compromised, and 81.5% reported two or more events of the disease.

The pair of nurses using the instrument containing the conceptual and operational definitions presented ICC above 0.80 for all the indicators assessed and four indicators presented absolute correlation (ρ=1.000). Note that all these correlations were statistically significant (Table 2).

**Table 2.** Intraclass correlation coefficient between the pair of nurses using conceptual and operational definitions and the pair not using definitions to measure the indicators concerning the nursing outcome Swallowing Status among patients affected by a stroke. (n=81). Fortaleza, CE, Brazil, 2013

Indicators*	With definitions			No definitions		
	ICC^†^	CI 95%‡	p-value	ICC^†^	CI 95%‡	p-value
1	1.000			0.405	0.206-0.571	˂0.001
2	1.000			0.123	-0.070-0.314	0.105
3	0.971	0.955-0.981	˂0.001	0.626	0.368-0.775	˂0.001
4	0.915	0.871-0.945	˂0.001	0.213	0.010-0.403	0.013
5	0.899	0.848-0.934	˂0.001	-0.016	-0.207-0.184	0.564
6	0.967	0.950-0.979	˂0.001	0.615	0.461-0.734	˂0.001
7	0.994	0.990-0.996	˂0.001	0.284	0.077-0.470	0.004
8	0.992	0.987-0.995	˂0.001	-0.071	-0.260-0.131	0.761
9	-	-	-	-	-	-
10	0.972	0.957-0.982	˂0.001	-0.021	-0.232-0.193	0.577

*Indicators: 1. Ability to bring food to mouth; 2. Postural Control of head and neck in relation to body; 3. Integrity of mastication structures; 4. Ability to maintain oral content in mouth; 5. Laryngeal elevation; 6. Discomfort when swallowing bolus; 7. Emptying the oral cavity after swallowing bolus; 8. Cough; 9. Regurgitation; 10. Respiratory aspiration. †CCI: Intraclass coefficient correlation; ‡CI 95%: Confidence Interval 95%.

For the pair of nurses who did not use the instrument with definitions, only five indicators presented significant correlation: Ability to bring food to mouth, Integrity of mastication structures, Ability to maintain oral content inside mouth, Discomfort when swallowing the food bolus and Emptying oral cavity after swallowing the bolus. Nonetheless, ICC (ρ<0.75) was low for all the indicators under study.

Note that in the assessment of the two pairs, the indicator Regurgitation did not present variance; that is, the two pairs assigned the same score to all the patients, as shown in Table 2.

All the indicators presented significant difference (p≤0.05) in the non-parametric analysis of variance using the Friedman test (Table 3). According to the *post hoc *intragroup comparison using the Minimal Important Difference calculation, the overall score was 0.535. Similarity was found between the pair using definitions when comparing the average ranks of the scores assigned by the nurses, both those using the definitions and those not using them, to measure the nursing outcomes (MID≤0.535). In turn, similarity within the pair using the instrument without definitions was not identified for Integrity of mastication structures and Ability to maintain oral content inside mouth (MID>0.535).

**Table 3.** Average ranks of scores assigned both by the nurses who used the conceptual and operational definitions and those who did not to measure the nursing outcome Swallowing Status among patients affected by a stroke. (n=81). Fortaleza, CE, Brazil, 2013

Indicators*	With definitions		Without definitions		p-value^†^
	1	2	1	2	
1	2.83	2.83	2.12	2.23	˂0.001
2	2.59	2.59	2.19	2.63	˂0.001
3	2.06	2.04	2.64	3.27	˂0.001
4	1.98	1.87	2.88	3.28	˂0.001
5	2.23	2.19	2.65	2.93	˂0.001
6	2.34	2.36	2.61	2.69	˂0.001
7	2.41	2.43	2.46	2.70	0.012
8	2.14	2.19	2.70	2.98	˂0.001
9	2.50	2.50	2.50	2.50	˂0.001
10	1.78	1.83	3.17	3.23	˂0.001

*Indicators: 1. Ability to bring food to mouth; 2. Postural Control of head and neck in relation to body; 3. Integrity of mastication structures; 4. Ability to maintain oral content in mouth; 5. Laryngeal elevation; 6. Discomfort when swallowing bolus; 7. Emptying the oral cavity after swallowing bolus; 8. Cough; 9. Regurgitation; 10. Respiratory aspiration. † Friedman test.

When performing intergroup comparison, MID was totally different (MID>0.535) for the indicators Ability to bring food to mouth, Integrity of mastication structures, Ability to maintain oral content inside mouth, and Respiratory aspiration. The MID method, however, did not identify differences in intergroup assessments for the indicators Postural control of head and neck in relation to body, Discomfort when swallowing bolus, and Emptying of oral cavity after swallowing bolus (Table 2).

Note that the differences between the assessments performed by the two pairs, in some situations, differed only for one of the nurses. According to the MID, evaluator 2 of the pair using the instrument containing the definitions and evaluator 1 from the pair with the instrument not containing the definitions presented similar assessments for the indicators Laryngeal elevation and Cough.

## Discussion

The use of definitions is essential for studies addressing nursing taxonomies such as NANDA International Inc. (NANDA-I) and the NOC because definitions fill in gaps between observation and clinical investigation^11^. In this case, in particular, the operational definitions describe what will be measured and how measurements can be performed.

One of the stages developed in this study was content validation. This practice was adopted due to a lack of a gold-standard^12^ with which to measure some phenomena of nursing interest. For this reason, assessment by judges with deep knowledge on the subject is essential.

A systematic review addressing instruments/items used to identify changes in the swallowing process or situations that favor aspiration, reports that blind inter-observer examination, the reapplication of the instrument, the use of indicators that do not generate doubt and minimal delay between physical assessment and checking of material, are priority activities that minimize differences among assessments^13^. Additionally, methodological rigor in the sequence of assessment procedures should be applied so that all the evaluators address assessment similarly^14^. This strategy requires operational definitions and shows that definitions make assessments more accurate.

In one study validating the conceptual and operational definitions of the nursing outcomes related to Ineffective Breathing Pattern of 45 children with congenital heart disease^11^, a lack of definitions was associated with inaccurate assessments concerning the patients' respiratory conditions. Note that in this study, the assessments concerning the indicators Ability to bring food mouth, Integrity of mastication structures, Ability to maintain oral content inside mouth, and Respiratory Aspiration performed by the pair not using the instrument with definitions totally disagreed with the assessments performed by the pair using the instrument with definitions.

The integrative review also shows that, in addition to the oral, pharyngeal and esophageal phases, there is the anticipatory phase, which is influenced by hunger, degree, and aspect of food, family environment, emotional state, social influences, use of utensils, hand-mouth coordination, and cervical posture. In addition to the need to observe swallowing, one has to pay attention to how food is handled on the plate, transported to the mouth, lip-closing, how food is manipulated inside the mouth and adjustment of trunk and head during feeding^15^. Therefore, the importance of verifying the indicators Ability to bring food to mouth and Postural control of head and neck in relation to body.

In regard to the indicator Regurgitation, no change was observed in this item in any of the patients. Note that what is reported in this respect is studies^15^^-^ 16, both in relation to the Concept Analysis and Content Validation. According to studies^15-16^ and the selected judges, only two signs can be observed when assessing regurgitation: its presence or absence. Hence, dividing into magnitudes (from 1 to 5) may have interfered in the results.

Another item that needs to be verified in a patient affected by a stroke is the strength of lip closure, because weak muscles lead to leaking of food out the side of the mouth^17^. In addition to this item, it is also necessary to assess the patient's ability to empty the oral cavity and the strength and symmetry of the palate^18^, voice quality, decrease or absence of cough, abnormal voluntary cough, cough while swallowing, pharyngeal elevation, and difficulty controlling salivation^13^^,^ 19 ^-^ 20_. _Therefore, verifying the indicators Integrity of mastication structures, Ability to maintain oral content, and Pharyngeal elevation, and cleaning of oral cavity after swallowing bolus all provide appropriate parameters to a structural and functional assessment of swallowing.

In addition to assessing aspects such as cough, anatomical and functional mastication structures, ability to wash away food and secretions and pharyngeal elevation, the clinical conditions related to risk of aspiration should also be investigated^21^. The presence of abnormal voluntary cough, abnormal vomiting reflex, change of voice, dysarthria, cough before/during/after swallowing and dysphonia are known clinical signs that indicate the presence of aspiration^13^^,^ 19. Hence, the indicator Respiratory aspiration, included after the Concept Analysis, ensured the investigation of these six clinical parameters.

Given what was observed, the use of definitions can help nurses to clinically assess many conditions present in patients after experiencing a stroke, especially to establish the correct magnitude of each indicator. In this way, it is possible to establish who is at the risk of aspiration in order to plan, early on, a neurorehabilitation program. Using instruments such as the one developed and validated in this study can increase the accuracy of these assessments.

## Conclusion

The results obtained by using the conceptual and operational definitions enabled establishing increased uniformity of clinical assessment of indicators of the nursing outcome Swallowing Status performed by nurses to ensure a more accurate result than when measurement was performed without using the definitions established for the indicators.

This study's limitations include the complex task of simultaneously gathering four evaluators to measure this nursing outcome in the clinical stage considering the dynamics of the healthcare facility under study. Additionally, even though the pairs using the instrument with definitions were trained equally, the potential for bias cannot be disregarded.

In addition, opting to work with a smaller number of judges in the content validation stage may have compromised the analyses performed; working with professionals from the multidisciplinary team, other than nurses, was difficult because these other professionals did not consider the concepts under study to be part of nursing science. Note that a lack of studies validating the NOC indicators for the outcome Swallowing Status, and especially nursing studies, with the use of psychometric and taxonomies limited the discussions of findings. For this reason, in some cases, the final concepts were developed based on expert opinion and the researchers' knowledge. 
